# Imaging and Methotrexate Response Monitoring of Systemic Inflammation in Arthritic Rats Employing the Macrophage PET Tracer [^18^F]Fluoro-PEG-Folate

**DOI:** 10.1155/2018/8092781

**Published:** 2018-02-21

**Authors:** Durga M. S. H. Chandrupatla, Gerrit Jansen, Elise Mantel, Philip S. Low, Takami Matsuyama, René P. Musters, Albert D. Windhorst, Adriaan A. Lammertsma, Carla F. M. Molthoff, Conny J. van der Laken

**Affiliations:** ^1^Amsterdam Rheumatology and Immunology Center, Location VUmc, VU University Medical Center, Amsterdam, Netherlands; ^2^Department of Chemistry, Purdue University, West Lafayette, IN, USA; ^3^Department of Immunology, Graduate School of Medical and Dental Sciences, Kagoshima University, Kagoshima 890-8544, Japan; ^4^Department of Physiology, VU University Medical Center, Amsterdam, Netherlands; ^5^Department of Radiology & Nuclear Medicine, VU University Medical Center, Amsterdam, Netherlands

## Abstract

**Background:**

In rheumatoid arthritis, articular inflammation is a hallmark of disease, while the involvement of extra-articular tissues is less well defined. Here, we examined the feasibility of PET imaging with the macrophage tracer [^18^F]fluoro-PEG-folate, targeting folate receptor *β* (FR*β*), to monitor systemic inflammatory disease in liver and spleen of arthritic rats before and after methotrexate (MTX) treatment.

**Methods:**

[^18^F]Fluoro-PEG-folate PET scans (60 min) were acquired in saline- and MTX-treated (1 mg/kg, 4x) arthritic rats, followed by tissue resection and radiotracer distribution analysis. Liver and spleen tissues were stained for ED1/ED2-macrophage markers and FR*β* expression.

**Results:**

[^18^F]Fluoro-PEG-folate PET and ex vivo tissue distribution studies revealed a significant (*p* < 0.01) 2-fold lower tracer uptake in both liver and spleen of MTX-treated arthritic rats. Consistently, ED1- and ED2-positive macrophages were significantly (*p* < 0.01) decreased in liver (4-fold) and spleen (3-fold) of MTX-treated compared with saline-treated rats. Additionally, FR*β*-positive macrophages were also significantly reduced in liver (5-fold, *p* < 0.005) and spleen (3-fold, *p* < 0.01) of MTX- versus saline-treated rats.

**Conclusions:**

MTX treatment reduced activated macrophages in liver and spleen, as markers for systemic inflammation in these organs. Macrophage PET imaging with [^18^F]fluoro-PEG-folate holds promise for detection of systemic inflammation in RA as well as therapy (MTX) response monitoring.

## 1. Introduction

Rheumatoid arthritis (RA) is a chronic inflammatory disease involving mainly the synovium of the joints, although other tissue/organ involvement has been recognized [1, 2]. Extra-articular manifestations occur in active and severe RA, including skin, eye, heart, lung, renal, nervous, and gastrointestinal systems [3, 4]. Therefore, early detection and treatment of systemically affected organs in RA could benefit in achieving predefined low disease activity and remission [5, 6]. To this end, in a preclinical setting, animal models of arthritis may serve a valuable tool for imaging (extra) articular and nonarticular inflammation and for monitoring the response to therapeutic interventions.

Many experimental animal models have been exploited to unravel the pathophysiology of inflammatory arthritis [7–12]. However, in most of these studies the primary research focus was on disease pathways and immune cells of the synovium rather than extra-articular manifestations. Also, depending on the modality and time frame of arthritis induction, extra-articular manifestations were not monitored or underreported.

Macrophages are known to play central role in RA disease progression [13]. Several studies have shown a direct correlation between disease remission and lower numbers of macrophage infiltration incidents into the synovium [14–16]. In patients, tissue resident macrophages in macrophage-rich organs such as liver and spleen may also be involved in extra-articular inflammation in RA [17]. Recent studies indicated that up to 50% of RA patients were reported with abnormal liver symptoms, including elevated alkaline phosphatase and small foci of necrosis and fatty liver [18]. Moreover, liver resident macrophages in an animal model were implicated in regulating chronic inflammation of arthritis through interacting with synovial phagocytes [19]. Not limiting to liver, spleen has also been reported in systemic inflammation in RA. Studies have shown manifestations of spleen enlargement and histological changes in either early or longstanding RA [20, 21].

Macrophage Positron Emission Tomography (PET) has been proposed as a noninvasive modality to monitor disease activity and therapy response in the whole body [22]. Beyond the prototypical macrophage tracer [^11^C]-PK11195, targeting the translocator protein (TSPO) on activated macrophages, second-generation TSPO tracers showed improved properties over [^11^C]-PK11195 to visualize arthritis [23]. Other interesting macrophage PET tracers to visualize arthritis are 4-[^18^F]-fluorophenylfolate, [^68^Ga]-DOTA-folate [24], and [^18^F]fluoro-PEG-folate [25]. These folate-based tracers bind with high affinity to folate receptor *β* (FR*β*) expressed on activated macrophages [26–28]. FR*β* is also of interest from a therapeutic perspective as it can bind and internalize antifolates and folate-conjugated antiarthritic therapeutics [26–31].

Recently we reported that the macrophage tracer [^18^F]fluoro-PEG-folate allowed visualizing arthritis in the inflamed knee joints of arthritic rats and also was able to monitor the response to the anchor drug in RA therapy, methotrexate (MTX) [32]. In the present study we extend on these observations by exploiting [^18^F]fluoro-PEG-folate PET to monitor potential systemic inflammation in liver and spleen of arthritic rats before and after MTX therapy, hypothesizing that MTX therapy also impacts systemic inflammatory effects in the organs. These studies were complemented with histological and immunofluorescence assessment of macrophage infiltration in liver and spleen.

## 2. Materials and Methods

### 2.1. Animals

The European community council directives 2010/63/EU for laboratory animal care and the Dutch law on animal experimentation criteria were fulfilled for performing the animal experiments. Wister rats (male, 150–200 grams, Charles River International Inc., Sulzfeld, Germany) were provided with standard food, water (ad libitum), and conditions as described previously [32]. The local committee on animal experimentation of the VU University Medical Center (DEC PET13-07) validated and approved experimental protocols.

### 2.2. Arthritic Induction and Therapeutic Interventions

Wistar rats were immunized [33] and arthritis was induced via 4x intra-articular (i.a.) methylated bovine serum albumin (mBSA) injections, 4 or 5 days apart in the arthritic (right) knee with the contralateral (left, nonarthritic) knee serving as control knee essentially as described before [33]. Rats were anesthetized during immunization and arthritic induction using inhalation anesthetics (isoflurane: 2–2.5% and oxygen: 1 L/min).

After the last i.a. injection the rats (*n* = 4/group) were treated 4x (d0, d7, d14, and d21) either with saline (500 *μ*L, intraperitoneal (i.p.) injection) or with MTX (VU University Medical Centers' Pharmacy) (i.p.) at 1.0 mg/kg. Healthy rats (nonarthritic) (*n* = 3) did not receive either arthritic induction or therapeutic interventions [32].

Six days after the last saline or MTX treatment, [^18^F]fluoro-PEG-folate PET scans were performed, immediately after which rats were sacrificed and tissues were excised for further processing and various analyses described hereafter.

### 2.3. [^18^F]Fluoro-PEG-Folate and PET

[^18^F]fluoro-PEG-folate was synthesized as previously described [25], with a radiochemical purity of >97% and mean specific activity of 49.7 ± 2.1 GBq/*μ*mol. Saline- and MTX-treated arthritic rats were anesthetized using inhalation anaesthetics (isoflurane: 2–2.5% and oxygen: 1 L/min). The jugular vein was cannulated with a polyurethane 3-French cannula (0.7 mm × 19 mm, BD Angiocath, Breda, Netherlands). During all procedures body temperature, heartbeat, respiratory rate, and blood oxygen saturation were monitored continuously using a rectal temperature probe and a pulse oxygen meter with SpO_2_ sensor. Anesthetized rats (*n* = 2, from saline- and MTX-treated groups) were positioned in a high resolution research tomograph (HRRT) (Siemens/CTI, Knoxville, TN, USA) and [^18^F]fluoro-PEG-folate (20.5 ± 3.4 MBq) was administered i.v. through the cannula and a dynamic PET scan was acquired for 60 min. Next, PET scans were normalized (for scatter, random, attenuation, decay, and dead time) and reconstructed as described before [25]. AMIDE software (version 0.9.2) [34] was used to analyse the images and data were expressed as standardized uptake values (SUV). The last frame was used to manually draw fixed size ellipsoidal shaped ROI over the area of liver and spleen (dimensions: 4 × 4 × 4 mm^3^) and arthritic and contralateral knees (dimensions: 7 × 4 × 7 mm^3^). The ROI for knees was drawn on top of the knee area [25] whereas, for liver and spleen, first a dotted line was drawn to represent the organ and then ROI was drawn approximately at the same spot in the saline- and MTX-treated rats. Through projecting ROIs onto the dynamic image sequence the time activity curve (TAC) was generated. TACs were expressed as standardized uptake values (SUV), that is, mean ROI radioactivity concentration normalized to injected dose and body weight.

### 2.4. Ex Vivo Tissue Distribution Studies

Rats (saline (*n* = 4), MTX (*n* = 4)) were sacrificed sixty minutes after [^18^F]fluoro-PEG-folate tracer administration [33]. Upon sacrificing, the knees, liver, and spleen were excised, rinsed, dipped dry, weighed, and the amount of radioactivity determined using an LKB 1282 Compugamma CS gamma counter (LKB, Wallac, Turku, Finland). Tissue radioactivity was expressed as percentage of the injected dose per gram tissue (%ID/g).

### 2.5. Histopathology and Immunohistochemistry

The liver and spleen sections from all rats (*n* = 3 for healthy rats and *n* = 4 for saline- and MTX-treated rats) were fixed in 4% neutral buffered paraformaldehyde for 24 h before embedding in paraffin wax. Sections of 5 *μ*m were cut and stained initially with haematoxylin and eosin and then with an ED1 (homologous to human CD68), ED2 (homologous to human CD163), or isotype control antibody [32]. ED2/CD163 serves as marker for M2-type (anti-inflammatory) macrophages. Images were captured using a Leica 4000B microscope and Leica digital camera DC500 (Microsystems B.V. Rijswijk, Netherlands).

### 2.6. FR*β* Immunofluorescence and Microscopy (Frozen Rat Tissue)

At the end of the study, liver and spleen tissues were collected from healthy rats (*n* = 3) and saline- and MTX-treated rats (*n* = 4) and snap frozen in liquid nitrogen and stored at −80°C. Tissues were embedded in appropriate media (OCT; SKU4583, Tissue-Tek, Netherlands) and were cut using cryotome (−20°C) (Leica, Netherlands) and placed on Superfrost (4951PLUS4, ThermoFisher, Netherlands) glass slides for immunofluorescence (IF) staining. Sections of 8 *μ*m were cut and stained with haematoxylin and eosin, and staining for FR*β*-positive macrophages was performed with a mouse anti-rat FR*β* antibody [29] or isotype control antibody.

For immunostaining, liver and spleen tissue sections were first brought to room temperature (RT) for 30 min, fixed in acetone (439126, Sigma-Aldrich, Netherlands) for 10 min at −20°C, and air-dried for 10 min at RT. A DAKO pen was used to mark the sections (S2002, DAKO, Santa Carla, CA, USA) which were subsequently washed 3x with PBS on a shaker. Next, sections were incubated with 100% fetal bovine serum (FBS) for 30 min at RT to avoid nonspecific binding and washed again in PBS (3 × 5 min). Thereafter, sections were incubated with mouse anti-rat FR*β* IgM (final concentration 1 *μ*g/ml) or isotype control IgM (ab35768, Abcam, Cambridge, UK; final concentration 1 *μ*g/ml) in 10% FBS/PBS for 24 hours at 4°C or with 10% FBS/PBS. After washing (3 × 5 min in PBS on a shaker), sections were incubated with goat-anti-mouse Alexa 488 ((final concentration 1 *μ*g/ml) (A21042) ThermoFisher Scientific, Netherlands) in 10% FBS/PBS, washed (3 × 5 min in PBS on a shaker), air-dried, and mounted with 2 *μ*l of MOWIOL mounting medium (81381, Merck, Zwijndrecht, The Netherlands). The 2D IF slides were imaged with a Zeiss Axiovert 200 M Marianas™ inverted microscope (40x oil-immersion lens). The microscope, camera, and data processing were controlled by SlideBook™ software (SlideBook version 6 (Intelligent Imaging Innovations, Denver, CO)) as described previously [35].

### 2.7. Quantification of Macrophages

The identity of all stained slides was hidden from and counted by two independent observers for FR*β* and ED1- and ED2-positive macrophages. For quantification, representative areas of liver and spleen sections were divided into 4 regions and counted at 400x magnification for FR*β* and ED1- and ED2-positive macrophages in the saline- and MTX-treated rats. The average numbers of macrophages per area from all four regions were combined and depicted as total numbers of FR*β*, ED1, or ED2 macrophages.

### 2.8. Statistical Analysis

Statistical analysis was performed using SPSS (version 15) for Windows (SPSS Inc., Chicago, IL, USA). Mann–Whitney (exact) tests were performed to analyse differences in tracer uptake (tissue distribution) macrophage infiltration in saline- versus MTX-treated groups. A *p* value < 0.05 was considered as statistically significant. All results are presented as mean ± standard deviation (SD).

## 3. Results and Discussion

### 3.1. Arthritis Induction and MTX Therapeutic Interventions

Upon arthritic induction all rats showed macroscopic thickening of the arthritic knee compared with the contralateral control knee (data not shown). As shown earlier, arthritis induction was well tolerated and allowed a window for therapeutic intervention with MTX, which was also well tolerated and not associated with any adverse effects [32].

### 3.2. [^18^F]Fluoro-PEG-Folate PET

**Figure 1:**
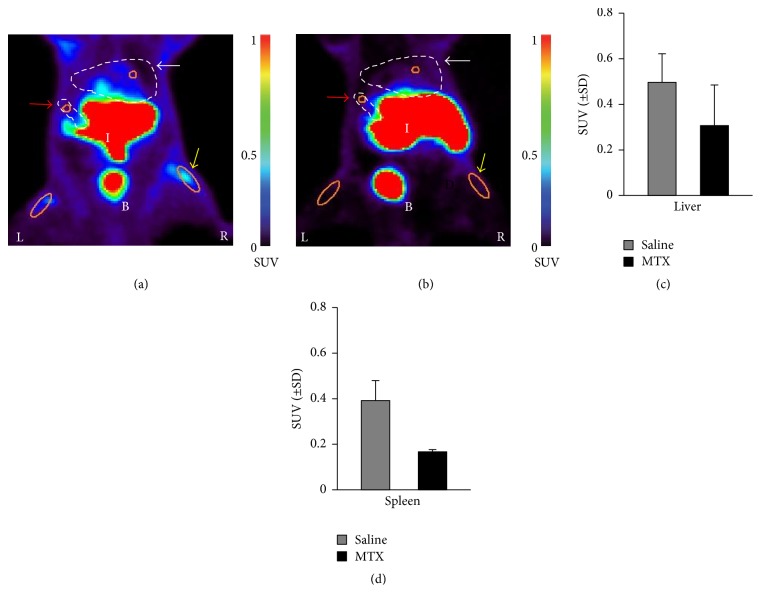
Representative coronal PET images of [^18^F]fluoro-PEG-folate in (a) saline-treated (*n* = 2) and (b) MTX-treated (*n* = 2) rats.* Orange ellipsoid*: ROI drawn around the synovium of the knee joint, liver (white arrow), spleen (red arrow), and arthritic* (right (R); yellow arrow)* and contralateral knees* (left (L))* depicted on each image. Spleen and liver areas are indicated by dashed lines. Standardized uptake value (SUV) scale bar from minimum 0 to maximum 1 represents the uptake of the tracer. Clearance organs intestine (I) and bladder (B) are also depicted. [^18^F]Fluoro*-*PEG*-*folate uptake is expressed as SUV (±SD) in (c) liver and (d) spleen of the saline- and MTX-treated group.

In a recent study we showed that imaging with the macrophage tracer [^18^F]fluoro-PEG-folate could visualize decreased accumulation of the tracer in the knee joints of arthritic rats treated with MTX. In the present study, we particularly focussed on macrophage-rich organs such as liver and spleen for their potential involvement in systemic inflammation and the impact of MTX therapy upon this. The coronal PET images visualized higher tracer uptake in liver and spleen of the saline-treated arthritic rats (Figure 1(a)) compared to the MTX-treated rats (Figure 1(b)). Standard uptake values (SUV) of [^18^F]fluoro-PEG-folate were quantified for liver and spleen with ROIs (colored ellipsoid) demonstrating decreased liver (1.5-fold) (Figure 1(c)) and spleen (2-fold) (Figure 1(d)) tracer uptake in MTX-treated compared to saline-treated rats. The relatively high uptake in the intestinal area, kidney, and bladder was due to the known clearance of the folate tracer [25, 32]. The MTX treatment results showed that, beyond knee joints, folate tracer binding is also inhibited by methotrexate in the extra-articular tissues, liver, and spleen, which suggests local anti-inflammatory effects on macrophage activity as part of systemic inflammation in these organs. These results are consistent with data from another arthritic rat model wherein [99^m^Tc]-EC20 folate scans also showed increased tracer uptake in liver and spleen [36] as compared to healthy rats. The increased tracer uptake in liver and spleen in arthritic rats coincided with increased tissue FR levels as measured by [^3^H]folic acid binding studies. Notably, a clinical study with [^18^F]-FDG, an indicator of active metabolism, in patients with collagen vascular disease-associated arthritis also showed significantly increased tracer uptake in the spleen, pointing to its inflammatory involvement [37]. In a clinical study in RA patients with [99^m^Tc]-EC20 folate, articular inflammation as well as liver and spleen involvement were demonstrated [38], further corroborating systemic inflammatory effects in arthritis.

### 3.3. Ex Vivo Tissue Distribution Studies

**Figure 2:**
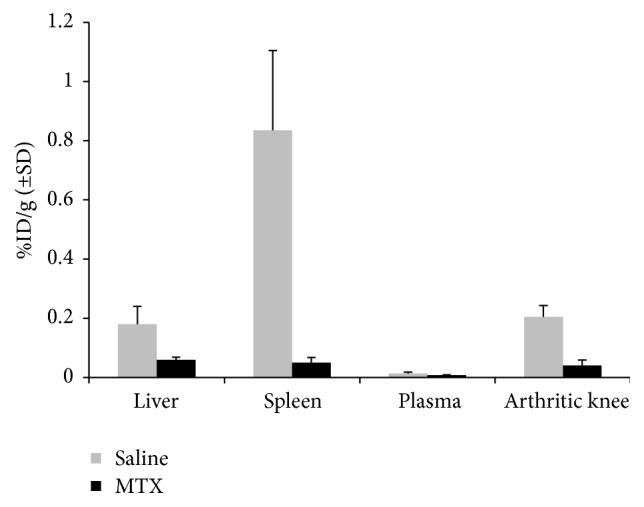
Ex vivo tissue distribution of [^18^F]fluoro*-*PEG*-*folate in liver, spleen, plasma, and arthritic knee of saline-treated (*n* = 4) and MTX-treated (*n* = 4) rats at 60 min after tracer injection. Results expressed as mean percentage injected dose per gram (%ID/g).* Error bars* indicate SD. *p* < 0.01 and *p* < 0.001.

To further establish the usefulness of therapeutic monitoring of systemic inflammation via [^18^F]fluoro-PEG-folate PET and regular MTX treatment (the anchor drug in RA), ex vivo tissue distribution studies were performed on selected tissues 60 minutes after tracer injection. In excised liver and spleen sections of MTX-treated rats, tracer uptake was significantly 3- and 16-fold lower (*p* < 0.01 and *p* < 0.001), respectively, compared to the saline-treated rats (Figure 2). For comparison, previously reported tracer uptake in liver and spleen of healthy rats was ~1.4-fold lower [32] than in arthritic rats also pointing at presence of systemic inflammation/macrophage activity in liver and spleen. The markedly lower tracer uptake (5-fold, *p* < 0.01) in the MTX-treated arthritic rat knees [32] is depicted as a reference (Figure 2). Plasma levels of [^18^F]fluoro-PEG-folate were low and comparable between both groups. Uptake of [^18^F]fluoro-PEG-folate in kidney (2.92 ± 0.33 versus 3.34 ± 0.63%ID/g) and intestine (1.06 ± 0.49 versus 0.84 ± 0/56%ID/g) is not significantly altered after MTX therapy. This is consistent with the notion that kidney constitutively expresses another FR isoform (i.e., FR*α*, implicated in renal retention of folates) [32].

It is of importance to note that tissue distributions data were obtained 6 days after the last MTX administration; thus it is unlikely that lowered tracer uptake is due to FR*β* blocking by MTX as residual plasma levels of MTX will be very low (<10 nM) at that stage [32]. Moreover, [^18^F]fluoro-PEG-folate binding affinity towards FR*β* outweighs MTX by at least 2-3 orders of magnitude [25, 27].

Together, PET and tissue distribution data illustrate that MTX treatment has a marked effect on macrophage tracer uptake in liver and spleen of arthritic rats.

### 3.4. Effect of MTX on Systemic Macrophage Infiltration

**Figure 3:**
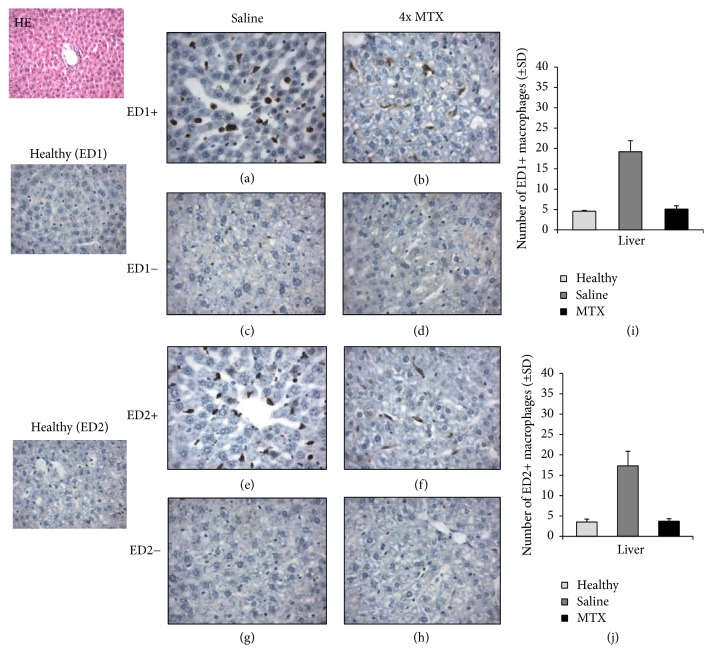
Representative immunohistochemical (HE) images of ED1^+^ and ED2^+^ macrophages in liver sections of healthy (*n* = 3) (ED1 and ED2), saline-treated (*n* = 4), and MTX-treated (*n* = 4) rats. ((a), (b)) Images represent ED1^+^ macrophages in the liver of saline-treated and MTX-treated rats, respectively. ((c), (d)) Isotype control stained liver sections of saline-treated and MTX-treated rats, respectively. ((e), (f)) Images of ED2^+^ macrophages in the liver of saline-treated and MTX-treated rats, respectively. ((g), (h)) Images of isotype control stained liver sections of saline-treated and MTX-treated rats, respectively. ((i), (j)) Bar graph representations of quantifications of ED1^+^ and ED2^+^ macrophages in liver of healthy, saline-treated, and MTX-treated rats. Values depict mean numbers of macrophages counted in predefined areas of the liver.* Error bars* indicate SD. *p* < 0.01.

**Figure 4:**
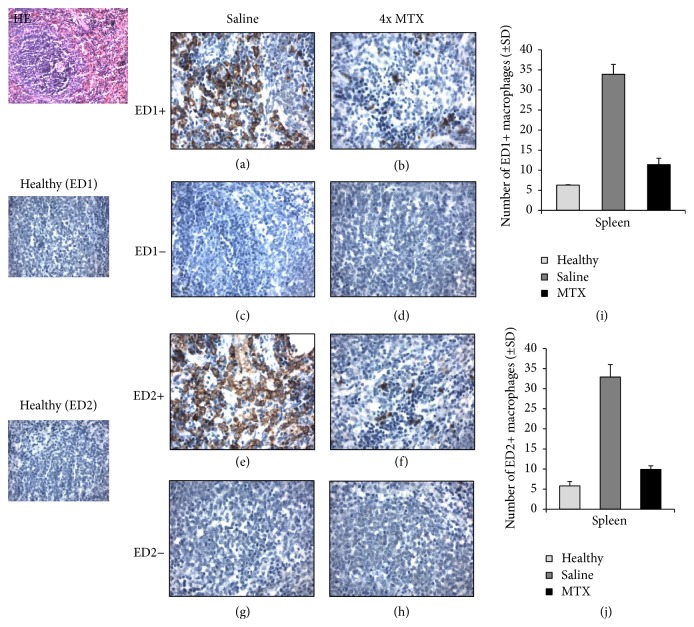
Representative immunohistochemical (HE) images of ED1^+^ and ED2^+^ macrophages in spleen sections of healthy (*n* = 3) (ED1 and ED2), saline-treated (*n* = 4), and MTX-treated (*n* = 4) rats. ((a), (b)) Images represent ED1^+^ macrophages in the spleen of saline-treated and MTX-treated rats, respectively. ((c), (d)) Isotype control stained spleen sections of saline-treated and MTX-treated rats, respectively. ((e), (f)) Images of ED2^+^ macrophages in the spleen of saline-treated and MTX-treated rats, respectively. ((g), (h)) Images of isotype control stained spleen sections of saline-treated and MTX-treated rats, respectively. ((I), (J)) Bar graph representations of quantifications of ED1^+^ and ED2^+^ macrophages in spleen of healthy, saline-treated, and MTX-treated rats. Values depict mean numbers of macrophages counted in predefined areas of the spleen.* Error bars* indicate SD. *p* < 0.01.

To extend on the PET and ex vivo tissue distribution data with [^18^F]fluoro-PEG-folate, the level of macrophage infiltration was examined in saline-treated and MTX-treated rats. Macrophage numbers were quantified in liver and spleen sections of saline-treated versus MTX-treated rats by immunohistochemical assessment of the abundance of total ED1-positive macrophages and ED2-positive macrophages, the latter, as CD163 homologue, serving as a proposed marker for anti-inflammatory macrophages. Figures 3 and 4 show representative images of ED1- and ED2-positive macrophages in liver and spleen sections. In liver and spleen of arthritic rats the numbers of ED1- and ED2-positive macrophages were ~5-fold higher (*p* < 0.01) than those of healthy rats.

For both ED1- and ED2-positive macrophages in liver and spleen, a marked decrease in macrophage infiltration is noted for MTX treatment compared to saline-treated rats. This was confirmed by a significantly (4-fold, *p* < 0.01) lower numbers of ED1- and ED2-positive macrophages in the liver of MTX-treated rats (Figures 3(i) and 3(j)), compared to saline-treated rats. Similarly, spleen sections of MTX-treated rats revealed significantly (3-fold, *p* < 0.01) lower quantifications of ED1- and ED2-positive macrophages, compared to saline-treated rats (Figures 4(i) and 4(j)). Antibody control stained liver and spleen sections were clearly negative for both ED1- and ED2-positive macrophages (Figures 3(c), 3(d), 3(g), 3(h), 4(c), 4(d), 4(g), and 4(h)). It is of interest to note that MTX impacted the infiltration of both ED1 and ED2 macrophage in liver and spleen of arthritic rats. For ED2 macrophages this may be counterintuitive given their assigned anti-inflammatory phenotype [13]. However, in the context of RA, recent evidence suggests that M2 macrophages can be skewed to produce proinflammatory cytokines [39], which can shift the balance of M2 to a more M1 phenotype. An alternative explanation could be that the MTX impacts circulating proinflammatory subsets of FR*β* expressing circulating monocytes [40] to suppress overall infiltration and polarization of macrophages in arthritic knees, liver, and spleen. Unravelling the exact mechanism of action of how MTX impairs macrophage infiltration awaits further research.

### 3.5. Effect of MTX on FR*β*-Positive Macrophages

FR*β*-positive synovial macrophages were shown to be highly infiltrated in the synovium of RA patients [27]. Given that [^18^F]fluoro-PEG-folate binds to FR*β* [25], we examined the expression of FR*β* in liver and spleen sections of saline-treated and MTX-treated arthritic rats to verify the data of the PET and tissue distribution studies. In liver and spleen of arthritic rats the number of FR*β*-positive macrophages was significantly (*p* < 0.01) higher than those of healthy rats.

**Figure 5:**
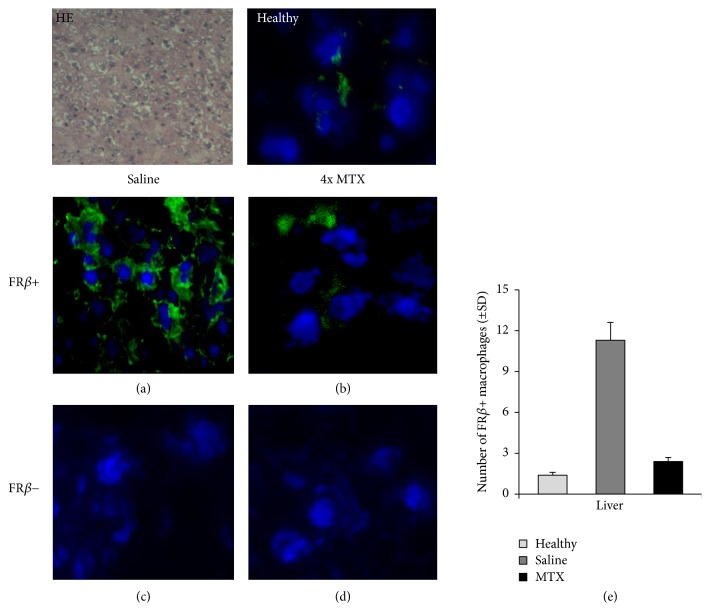
Representative immunofluorescence images of FR*β*^+^ macrophages in liver sections of healthy (*n* = 3) and saline- (*n* = 4) and MTX-treated (*n* = 4) rats. ((a), (b)) Images represent FR*β*^+^ macrophages in the liver of saline-treated and MTX-treated rats, respectively. ((c), (d)) Isotype control stained liver sections of saline-treated and MTX-treated rats, respectively. (e) Bar graph representation of quantifications of FR*β*^+^ macrophages in liver of saline-treated and MTX-treated rats. Values depict mean numbers of macrophages counted in predefined areas of the liver.* Error bars* indicate SD (blue color: DAPI (nucleus staining); green color: FR*β* staining). *p* < 0.01.

**Figure 6:**
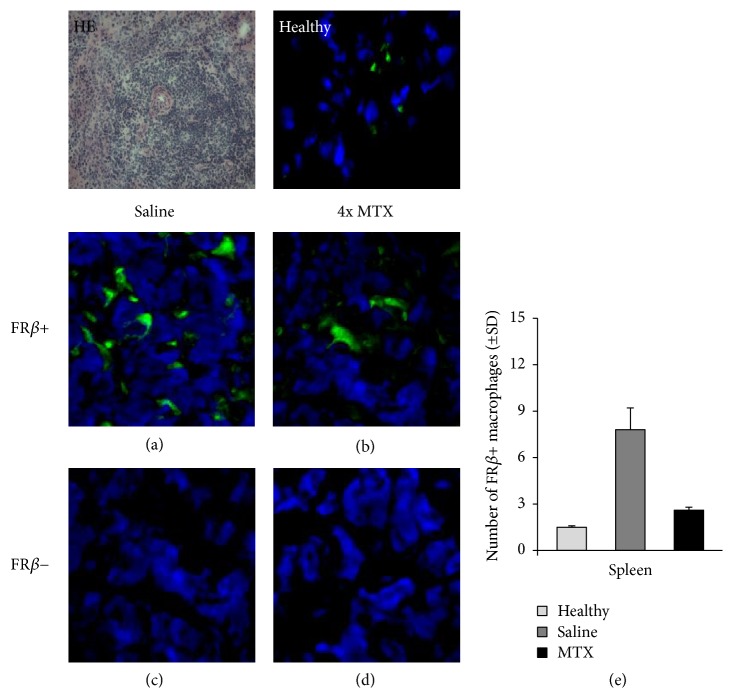
Representative immunofluorescence images of FR*β*^+^ macrophages in spleen sections of healthy (*n* = 3) and saline- (*n* = 4) and MTX-treated (*n* = 4) rats. ((a), (b)) Images represent FR*β*^+^ macrophages in the spleen of saline-treated and MTX-treated rats, respectively. ((c), (d)) Isotype control stained spleen sections of saline-treated and MTX-treated rats, respectively. (e) Bar graph representation of quantifications of FR*β*^+^ macrophages in spleen of saline-treated and MTX-treated rats. Values depict mean numbers of macrophages counted in predefined areas of the spleen.* Error bars* indicate SD. (blue color: DAPI (nuclear staining); green color: FR*β* staining). *p* < 0.01.

Representative immunofluorescence images of FR*β* expression in cryosections of liver (Figures 5(a)–5(d)) and spleen (Figures 6(a)–6(d)) after saline and MTX therapeutic interventions revealed a markedly lower FR*β* expression in both liver and spleen of MTX-treated versus saline-treated rats. This was confirmed by quantitative assessments showing significant 5-fold (*p* < 0.005) and 3-fold (*p* < 0.01) lower numbers of FR*β*-positive macrophages in the liver (Figure 5(e)) and spleen (Figure 6(e)) of MTX-treated rats. The FR*β* levels in MTX-treated rats approximated FR*β*-positive macrophages in liver and spleen of healthy rats. Antibody control stained liver and spleen sections were negative (Figures 5(c), 5(d), 6(c), and 6(d)). Results for FR*β* staining were consistent with ED1 and ED2 stainings (Figures 3 and 4). Together, these results underscore that macrophage infiltration in liver and spleen is implicated in inflammation and response to therapy, similar to that shown for RA synovium in patients [14–16].

In addition to MTX, antiarthritic effects elicited through FR*β* targeting have been reported for folate-conjugated immunotoxins [29] and various folate-conjugated drugs [30, 41, 42]. Since FR*β* is primarily expressed on activated macrophages [27, 28], microenvironmental conditions in liver and spleen will be of importance for FR*β* expression and macrophage polarization. FR*β* expression has been reported on both M1- and M2-type macrophages [43], and in rat RA synovium FR*β* expression has been also observed on a mixed M1- and M2-type [44]. As indicated above, in the RA microenvironment with circulating complex IgG autoantibodies and/or ACPA antibodies, FR*β* expressing activated macrophages can release proinflammatory cytokines [39, 45] and thus be a bona fide target. More detailed investigations on the specific polarization and phenotypic properties of FR*β*-expressing tissue macrophages in liver and spleen may assist optimal targeting of this receptor for imaging and therapeutic exploitations. These premises do not only hold for arthritis but also for cancer [46].

## 4. Conclusion

MTX treatment reduced activated macrophages in liver and spleen, as markers for systemic inflammation in these organs. Macrophage PET imaging with [^18^F]fluoro-PEG-folate holds promise for detection of systemic inflammation in RA as well as therapy (MTX) response monitoring.

## Abbreviations

RA: Rheumatoid arthritis
mBSA: Methylated bovine serum albumin
MTX: Methotrexate
HRRT: High resolution research tomography
PEG: Polyethylene glycol
SD: Standard deviation
%ID/g: Percentage of the injected dose per gram
i.a.: Intra-articular
FR*β*: Folate Receptor *β*
ROI: Region of interest
EC20: A folate-linked chelator of 99mTc.
